# Risk Prediction Models for Invasive Mechanical Ventilation in Patients with Autoimmune Encephalitis: A Retrospective Cohort Study

**DOI:** 10.1155/2023/6616822

**Published:** 2023-12-05

**Authors:** Shiyang Xie, Meilin Chen, Luying Qiu, Long Li, Shumin Deng, Fang Liu, Hefei Fu, Yanzhe Wang

**Affiliations:** ^1^Department of Radiation Oncology, The First Hospital of China Medical University, Shenyang, China; ^2^Innovation Center for Neurological Disorders and Department of Neurology, Xuanwu Hospital, Capital Medical University, Beijing, China; ^3^Department of Neurology, Key Laboratory for Neurological Big Data of Liaoning Province, The First Affiliated Hospital of China Medical University, Shenyang, China; ^4^Department of Neurosurgery, The First Hospital of China Medical University, Shenyang, China

## Abstract

**Methods:**

A multivariate predictive nomogram model was developed using the risk factors identified by LASSO regression and assessed by receiver operator characteristics (ROC) curve, calibration curve, and decision curve analysis.

**Results:**

The risk factors predictive of severe respiratory failure were male gender, impaired hepatic function, elevated intracranial pressure, and higher neuron-specific enolase. The final nomogram achieved an AUC of 0.770. After validation by bootstrapping, a concordance index of 0.748 was achieved.

**Conclusions:**

Our nomogram accurately predicted the risk of developing respiratory failure needing IMV in AE patients and provide clinicians with a simple and effective tool to guide treatment interventions in the AE patients.

## 1. Introduction

Autoimmune encephalitis (AE) is a severe neurological brain disorder that occurs when the immune system attacks the nervous tissue within the brain. This disease can cause mild, temporary symptoms such as memory loss and altered consciousness. However, in some cases, AE can cause severe symptoms that require prolonged intensive care (ICU) treatment. These symptoms may include diminished consciousness, severe dyskinesia, autonomic dysfunction, epileptic seizures, coma, or severe respiratory failure requiring invasive mechanical ventilation (IMV) [1–3]. Due to the severity of these symptoms, studies have shown that AE can lead to death in about 2.3%–9.5% of patients [4–6].

Acute respiratory failure requiring IMV remains a common occurrence during the acute phase of this life-threatening illness and is one of the most frequent medical justifications for ICU admissions in AE patients. Patients at risk of developing severe respiratory failure can be treated with elective intubation. However, patients that present with severe respiratory failure may require emergency intubation. Emergency intubation can increase the risk of complications after treatment and often leads to worse clinical outcomes [7, 8]. Therefore, early detection of patients at risk of severe respiratory failure may allow clinicians to implement therapeutic measures in a timely manner, thereby improving the prognosis.

Various factors are known to increase the risk of developing severe respiratory failure requiring ICU in AE patients. These factors include anemia [9], a white blood cell (WBC) count in the cerebrospinal fluid (CSF) above 20 cells/mm^3^ [10], failure to respond to first-line immunotherapy, and high-interleukin-17A (IL-17A) concentrations in the CSF [11, 12]. However, to date, there is no standard prediction model that comprehensively includes both the clinical symptom severity and laboratory tests. In this study, we aimed to develop a simple nomogram to assess the risk of developing severe respiratory failure requiring IMV in AE patients-based solely on readily available clinical characteristics.

## 2. Materials and Methods

### 2.1. Inclusion and Exclusion Criteria

Patients treated for AE between January 2017 and December 2021 at the First Affiliated Hospital of China Medical University were eligible for this study. The patients were included in this study if they were diagnosed with AE according to the criteria established by Graus et al. [13], had antibodies related to AE detected in the serum and or CSF by cell-based assay (CBA), an indirect immunofluorescence (IIF) test, or immunospot assay and serum and CSF albumin levels analyzed simultaneously before immunotherapy. Patients with other neural antibodies and those diagnosed with neurological or psychiatric disorders, including but not limited to ischemic or hemorrhagic stroke, intracranial tumors, intracranial infection, toxic-metabolic encephalopathy, epilepsy, and schizophrenia, were excluded from the study. Patients were also excluded if they did not complete the final outcome interview, had missing baseline information, and if they received IMV treatment before hospitalization.

### 2.2. Clinical Data Collection

**Figure 1:**
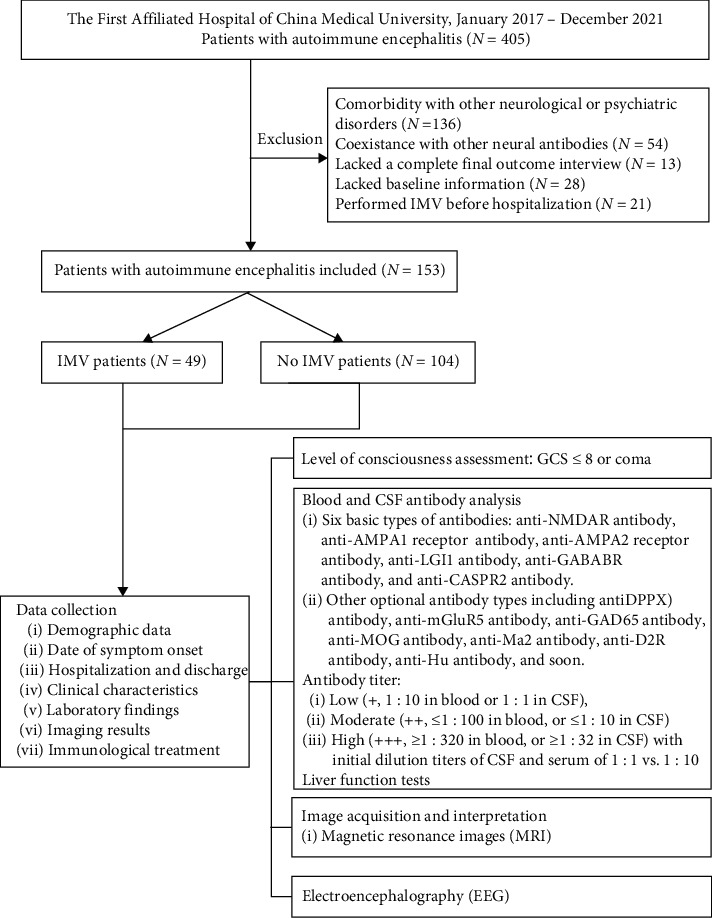
Flowchart illustrating the data-collection process.

Three neurologists extracted the demographic data (gender, age), date of symptom onset, hospitalization and discharge, clinical characteristics (comorbidities, level of consciousness, clinical symptoms such as seizures, psychiatric symptoms, and memory impairment at the onset of the diagnosis, throughout the hospitalization period, and during discharge), laboratory findings (CSF test, blood test, and liver function test), imaging results, and the immunological treatment provided were extracted from the patient's medical records. The study flowchart is shown in Figure 1.

#### 2.2.1. Level of Consciousness Assessment

The Glasgow Coma Scale (GCS) score was used to determine patients' level of consciousness at admission. A GCS score of 8 or coma at admission indicated a consciousness disorder [14, 15].

#### 2.2.2. Blood and CSF Analysis

The serum and CSF samples were simultaneously collected at the acute AE stage before immunotherapy. Lumbar puncture opening pressure measurements were also used to assess intracranial pressure (ICP). The leukocyte count, total protein, glucose levels, chlorine ions, and color status were evaluated to assess the physicochemical characteristics of CSF. The concentration of antibodies related to AE was measured in the serum and CSF.

Liver function tests were also performed. Patients with alanine aminotransferase (ALT) or aspartate aminotransferase (AST) levels three times the normal upper limit or bilirubin levels twice the normal upper limit was identified as impaired hepatic function [16].

#### 2.2.3. Image Acquisition and Interpretation

Magnetic resonance images (MRI) were acquired using a 1.5 T scanner during the acute AE stage. One skilled neurologist independently assessed the images. New-onset brain lesions with aberrant signals on T1-weighted, T2-weighted, fluid-attenuated inversion recovery (FLAIR), diffusion-weighted, or contrast-enhanced T1-weighted images were characterized as brain MRI abnormalities.

#### 2.2.4. Electroencephalography (EEG)

An EEG was acquired on admission. Any EEG abnormalities were defined as moderate or severe.

### 2.3. Statistical Analysis

The categorical variables were represented as counts and percentages, while the continuous variables were represented as means (standard deviations (SD)) or medians (interquartile ranges). The patients were then divided into two groups depending on whether they required IMV treatment (IMV group) on not (non-IMV group). The categorical variables between the IMV group and non-IMV group were compared using Fisher's exact test or the Pearson *χ*^2^ test (*χ*^2^). Conversely, the Student's *t*-test or the Mann–Whitney *U* test was used to compare the continuous variables. The least absolute shrinkage and selection operator (LASSO) method was used to identify the risk factors for developing severe respiratory failure requiring IMV [17]. The nonzero variables identified by the minimum lambda in the LASSO regression were included in a multivariate logistic regression. The multivariate regression analysis was used to measure the odds ratios (OR) at the 95% confidence interval (95%CI). The *P*-value of <0.05 for two-tailed was regarded as statistically significant. The significant risk factors identified by the multivariate logistic regression were finally included in the nomogram. The nomogram assigns a risk score for each identified clinical risk factor. Points were assigned to the nomogram by drawing a vertical line from each predictor-associated value to the axis points. The total risk of developing severe respiratory failure was then calculated by summing up all scores. All potential predictors were used to construct the predictive nomogram.

A calibration plot was used to assess the concordance index (C-index) of the nomogram. Due to the relatively small sample, the nomogram was internally validated using the bootstrapping method (1,000 bootstrap resamples). The area under the curve (AUC) of a receiver operator characteristics (ROC) curve was used to determine the discrimination accuracy of the model [18]. Finally, the model's net clinical benefit was evaluated using a decision curve analysis at various threshold probabilities [19].

## 3. Results

### 3.1. Clinical Characteristics of AE

The data-collection process is shown in Figure 1. A total of 405 patients were treated with AE between January 2017 and December 2021 at the First Affiliated Hospital of China Medical University, of whom 13 were excluded because they did not complete the final outcome interview, 28 had missing baseline information, and 21 were treated with IMV before being hospitalized. Of the 153 enrolled patients, 32% needed IMV. All patients tested positive for neuronal antibodies, including antibodies against NMDAR, LGI1, CASPR2, GABA_B_R, AMPA, GAD65, and MOG.  Table 1 provides a summary of the patient's characteristics. For most patients (58.8%, *n* = 90), the time between the onset of symptoms and hospitalization was less than a month. All patients were admitted to the hospital during the acute phase of the disease. On admission, short-term memory dysfunction was the most common clinical symptom, followed by epilepsy. Approximately 90.2% of patients were treated with corticosteroids, 32% received intravenous immunoglobulin, and 6% were administered immunosuppressive agents.

### 3.2. Development of the Individualized Prediction Model

The baseline characteristics of patients requiring IMV and those that did not require IMV on admission are summarized in Table 1. The patients in the IMV group were more likely to be male and presented with more clinical symptoms such as cognitive impairment, short-term memory dysfunction, calculating dysfunction, consciousness disorders, language dysfunction, and extrapyramidal symptoms than the patients in the non-IMV group (*P* < 0.05). The incidence of patients with raised ICP (*P*=0.04), impaired hepatic function (*P* < 0.001), pulmonary infections (*P* < 0.001), and abnormal MRI results (*P*=0.002) was also significantly higher in the IMV group than that of the non-IMV group. Moreover, patients in the IMV group had higher CSF antibody titer values (*P*=0.001), CSF cell count (*P*=0.025), C-reactive protein (CRP) (*P*=0.001), erythrocyte sedimentation rate (ESR) (*P*=0.015), and lower levels of free triiodothyronine (FT3) (*P*=0.042).

**Figure 2:**
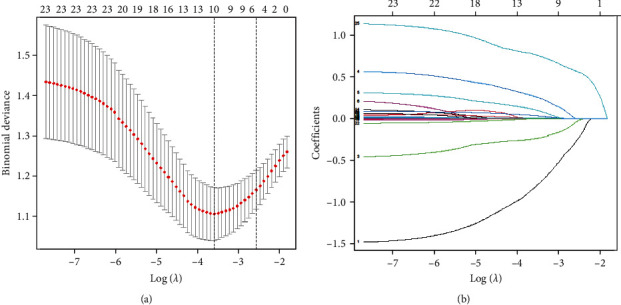
Significant clinical risk factors identified by the least absolute shrinkage and selection operator (LASSO) logistic regression model. (a) Minimum criterion (*λ*. min) and the one standard error (SE) of the minimum criterion (*λ*. 1se). The dashed vertical line indicates the optimal value. (b) The LASSO synergistic analysis of 25 variables. The curve coefficients were plotted according to a logarithmic series. The best predictor of the model was determined by tenfold cross-validation of the minimum criterion. The optimal *λ* was defined as the value that produces nonzero coefficients for 10 features.

The optimal lambda (*λ*) value for the LASSO was 0.02742195. Ten clinical features had nonzero coefficients, including gender, antibody subtype, antibody titers in blood, antibody titers in the CSF, liver function, chloride level in CSF, intracranial pressure (ICP), CRP, ESR, and NSE (Figure 2). These clinical features were included in the multivariate analysis, as shown in Table 2. The multivariate analysis identified male gender (*P*=0.003), impaired hepatic function (*P*=0.047), raised ICP (*P*=0.031), and elevated NSE (*P*=0.047) as independent predictors for IMV.

### 3.3. Performance of the Prediction Nomogram

**Figure 3:**
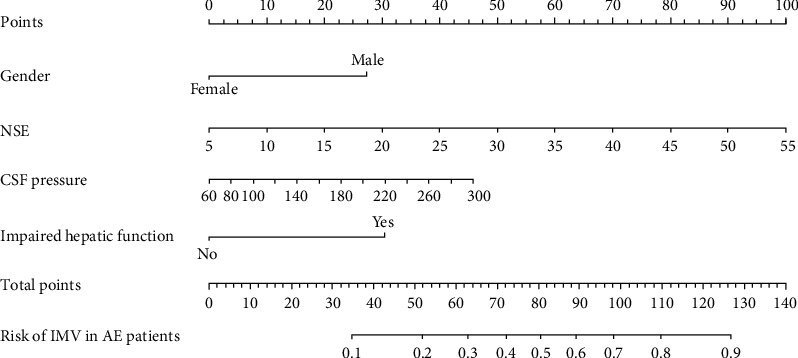
Nomogram predicting the risk of needing invasive mechanical ventilation (IMV) in patients with autoimmune encephalitis (AE).

**Figure 4:**
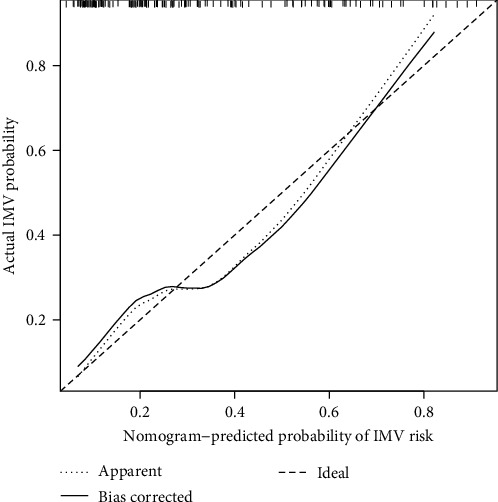
Calibration curves assessing the accuracy of the predictive nomogram.

**Figure 5:**
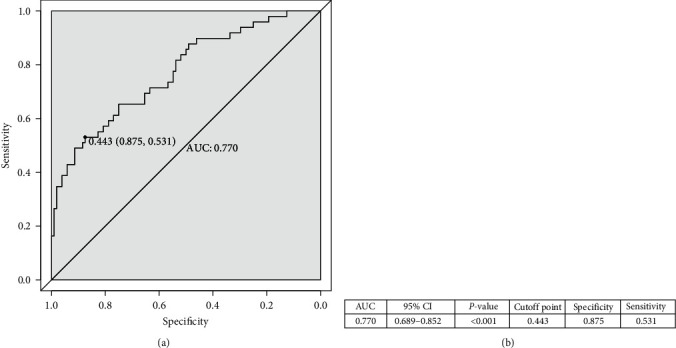
(a) Receiver operator characteristics (ROC) curve for the predictive nomogram. (b) The values of ROC.

**Figure 6:**
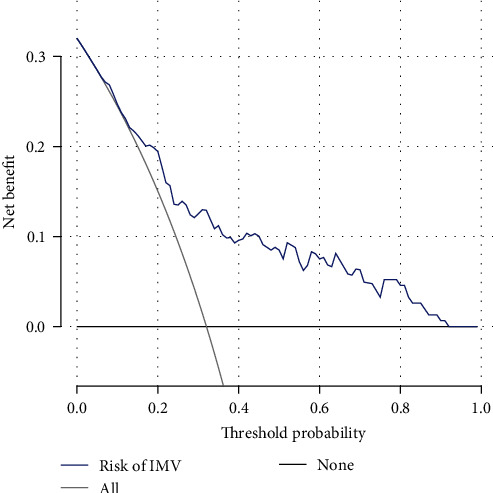
Decision curve analysis used to evaluate the clinical benefit of the predictive nomogram.

The nomogram was produced using the most significant predictors identified in the multivariate analysis (Figure 3). The bootstrapped nomogram calibration curves achieved a C-index of 0.748, and all prediction probabilities were either on or near the 45° line of the plot (Figure 4). These findings indicate a good agreement between the actual and the forecasted nomogram results. The AUC of the nomogram was 0.770 (95% CI, 0.689–0.852), suggesting that the nomograms for the cohort had a good prediction accuracy (Figure 5). The decision curve analysis for the nomogram, which was used to evaluate the model's clinical applicability, is shown in Figure 6. The population's risk cutoff, below which patients would see a therapeutic benefit, ranged from 5% to 91%. Based on the IMV risk nomogram, the net benefit within this range was comparable with some overlaps.

## 4. Discussion

Patients with AE often develop severe respiratory symptoms that require IMV treatment. Failure to identify the risk of developing severe respiratory symptoms at an early stage can lead to prolonged recovery and even death. However, few studies have evaluated the factors that may increase the severity of AE except in cases of anti-NMDAR encephalitis. Therefore, in this study, we evaluated the clinical factors in a cohort of 153 antibody-confirmed AE patients that could increase the risk of needing IMV. Nomograms are increasingly being used as prognosis prediction tools to facilitate clinical decision-making [20]. However, to our knowledge, no IMV risk prediction models are currently available for AE patients. Therefore in this study, we developed a risk prediction nomogram for IMV based on easily available clinical variables that could be used by doctors to facilitate decision-making.

This study identified four clinical risk factors for IMV: male gender, impaired hepatic function, raised ICP, and elevated NSE. In line with the previous studies, we found that males were more likely than females to develop severe respiratory failure. A study using an AE mouse model found that male mice had a greater risk of developing severe neurodegeneration than female mice [21]. Although the exact cause for the higher incidence of severe AE in men is still unknown, May et al. [22] suggested that estrogen in women can enhance the function of the blood–brain barrier by increasing the function of the inter-endothelial cells and by limiting the transmigration of lymphocytes via the regulation of the downstream protein. Furthermore, sexual hormones may also play a role in the progression of autoimmune disorders.

Raised ICP is typically associated with mass effects caused by space-occupying lesions. However, central nervous system infections, such as meningitis or encephalitis, can also increase the ICP [23]. Regardless of the level of pleocytosis or protein, an increased ICP should always be considered when predicting the risk of needing IMV. It is possible that the way in which this variable was analyzed contributed to the ambiguity concerning the impact of CSF abnormalities on anti-NMDAR encephalitis [24]. de Montmollin et al. [25] found that a lower CSF white blood cell count was associated with better functional outcomes. However, this study used a very high-cutoff point (50 cells/mm^3^) to distinguish between inflammatory and noninflammatory CSF lesions, while most studies typically used a lower cutoff point (5 cells/mm^3^) [25]. Similarly, when examining the CSF protein levels, similar methodological considerations must be considered [26]. Few studies evaluated the incidence and clinical significance of elevated ICP in patients with AE. Thus, further research is needed to understand better the relation between ICP and the development of severe respiratory failure.

The hepatic function had a minor impact on the risk of developing severe respiratory infection. However, it was still included in the nomogram. Hepatic function was frequently assessed as part of routine laboratory examinations, but this factor was not identified as a prognostic factor in AE. However, studies have shown that patients with autoimmune diseases can have elevated transaminase, and in rare cases, patients can develop idiosyncratic and unpredictable acute liver failure during immunosuppressive therapy [27]. Therefore abnormal hepatic function can serve as an early indicator of critical illness in AE patients. Furthermore, the clinician must monitor any clinical symptoms indicative of liver involvement, particularly during immunosuppressant treatment.

In our study, NSE was also linked to a higher risk of needing IMV in AE patients. The NSE enzyme regulates glycolysis and is expressed in many neurons and neuroendocrine cells. High levels of NSE indicate neuronal damage [28]. Higher NSE was also noted in patients suffering from obstructive sleep apnea [29]. Therefore, dysregulation of the immune system can damage the central nervous system and ultimately lead to the respiratory failure.

Unexpectedly, no correlation was found between antibody titers and the risk of developing severe respiratory failure. In addition, there was no clear relationship between the clinical severity and serum antibody titers. Consistent with the previous studies, certain relapsing antibodies were only found in the CSF [30, 31]. Therefore the antibody titers were not included in the nomogram. However, it is important to note that in our study, patients without autoantibodies in serum or CSF have been classified as potential AEs rather than as definite AEs as indicated by the Graus criteria. Potential AE patients were excluded from our study.

Our nomogram based on the four main clinical risk factors achieved high-predictive accuracy and overall net clinical benefit. This tool can easily be implemented in the department. The evaluation process for IMV can be divided into three steps. The first step should include a physical examination with special attention to the respiratory symptoms. The presence of dyspnea may be a sign of pulmonary infections and exudative changes, which may aggravate the gas exchange function. In the second step, a blood test should be performed to assess liver function and NSE levels. Finally, the ICP should be assessed by performing a lumbar puncture.

Our study has several limitations that have to be acknowledged. First of all, the retrospective nature of this study could lead to information bias due to its inability to control confounding factors. In addition, the data were collected from a single center which could potentially limit the generalizability of the findings.

## 5. Conclusion

Male gender, impaired hepatic function, raised ICP, and elevated NSE were identified as independent risk factors for developing severe respiratory failure that requires IMV. These factors are easy to obtain clinically and were therefore included in the predictive nomogram. Our nomogram accurately predicted the risk of developing respiratory failure needing IMV in AE patients. This model could provide an effective tool for clinicians to optimize treatment interventions in the AE patients. However, external validation is required to confirm the generalisability of the model.

## Figures and Tables

**Table 1:** Characteristics of patients with AE.

Characteristics	All	No IMV patients	IMV patients	*P*-value
	(*N* = 153)	(*N* = 104)	(*N* = 49)	
Gender (male/female)	83/70	65/39	18/31	0.003
Age, years	50.22 ± 17.49	51.18 ± 15.91	48.18 ± 18.59	0.324
Positive antibody, *n* (%)				<0.001
NMDAR	53 (34.6)	31 (29.8)	22 (44.9)	
GAD 65	6 (3.9)	6 (5.8)	0 (0)	
LGI1	52 (33.9)	44 (42.3)	8 (16.3)	
GABA_B_R	25 (16.3)	12 (11.5)	13 (26.5)	
AMPAR	6 (3.9)	0 (0)	6 (12.2)	
CASPR2	6 (3.9)	6 (5.8)	0 (0)	
MOG	5 (3.3)	5 (4.8)	0 (0)	
Antibody subtype, *n* (%)				<0.001
Anti-intracellular antigen	6 (3.9)	6 (5.8)	0 (0)	
Anti-synaptic receptors	84 (54.9)	43 (41.3)	41 (83.7)	
Anti-ion channels & other surface proteins	63 (41.2)	55 (52.9)	8 (16.3)	
Clinical Subtype, *n* (%)				0.024
Anti-NMDAR encephalitis	53 (34.6)	31 (29.8)	22 (44.9)	
Limbic encephalitis	89 (58.2)	62 (59.6)	27 (55.1)	
Other AE syndrome	11 (7.2)	11 (10.6)	0 (0)	
Blood CBA, *n* (%)				0.054
negative	21 (13.7)	18 (17.3)	3 (6.1)	
+	72 (47.1)	47 (45.2)	25 (51.0)	
++	36 (23.5)	27 (26.0)	9 (18.4)	
+++	24 (15.7)	12 (11.5)	12 (24.5)	
CSF CBA, *n* (%)				<0.001
Negative	11 (7.2)	6 (5.8)	5 (10.2)	
+	9 (5.9)	9 (8.7)	0 (0)	
++	43 (28.1)	38 (36.5)	5 (42.9)	
+++	90 (58.8)	51 (49.0)	39 (46.9)	
Clinlcal symptom, *n* (%)				
Epilepsy	128 (83.7)	87 (83.7)	41 (83.7)	1.000
Short-term memory dysfunction	129 (84.3)	81 (77.9)	48 (98.0)	0.001
Calculation dysfunction	122 (79.7)	75 (72.1)	47 (95.9)	<0.001
Psychiatric symptoms	91 (59.4)	58 (55.8)	33 (67.3)	0.217
Consciousness disorders	21 (13.7)	4 (3.8)	17 (34.7)	<0.001
Language dysfunction	84 (54.9)	50 (48.1)	34 (69.4)	0.015
Extrapyramidal symptoms	41 (26.8)	22 (21.2)	19 (38.8)	0.031
Autonomic dysfunction	53 (34.6)	35 (33.7)	18 (36.7)	0.719
Interval from symptoms onset to hospital admission (months), *n* (%)				0.216
≤1	90 (58.8)	59 (56.7)	31 (63.3)	
1–3	43 (28.1)	28 (26.9)	15 (30.6)	
>3	20 (13.1)	17 (16.3)	3 (6.1)	
Thyroid function tests				
TPOAb, *n* (%)	43 (28.1)	27 (26.0)	16 (32.7)	0.442
TGAb, *n* (%)	54 (35.3)	34 (32.7)	20 (40.8)	0.367
FT3 (2.63–5.7) pmol/L	3.58 ± 1.04	3.70 ± 3.33	3.33 ± 0.93	0.042
FT4 (9.01–19.05) pmol/L	13.22 ± 2.59	13.21 ± 2.26	13.23 ± 3.21	0.974
TSH (0.35–4.94) mlU/L	1.73 ± 2.08	1.82 ± 2.04	1.54 ± 2.16	0.456
CSF test				
ICP (80–180) mmH_2_O	164.61 ± 60.96	153.70 ± 51.89	187.76 ± 72.03	0.004
CSF protein (120–600) mg/L	622.15 ± 452.25	600.52 ± 400.27	668.08 ± 535.56	0.390
CSF Glucose (2.2–3.9) mmol/L	3.79 ± 1.06	3.82 ± 1.00	3.72 ± 1.19	0.593
CSF Cl (120–132) mmol/L	119.71 ± 5.53	119.23 ± 4.97	120.72 ± 6.51	0.120
CSF cell (0–15) ^*∗*^10^9^/L	44.12 ± 83.19	33.78 ± 86.24	66.08 ± 73.32	0.025
Blood tests				
CRP (0–6) mg/L	30.14 ± 50.68	19.67 ± 41.9	52.36 ± 60.21	0.001
ESR (0–20) mm/h	18.24 ± 18.13	15.59 ± 15.46	23.86 ± 20.31	0.015
CEA (0–4.3) ng/mL	3.28 ± 3.56	3.25 ± 3.96	3.34 ± 2.56	0.884
AFP (0–7) ng/mL	2.85 ± 1.62	2.95 ± 1.63	2.63 ± 1.60	0.248
CA125 (0–35) U/mL	16.52 ± 18.50	15.78 ± 21.12	18.07 ± 11.07	0.476
CA153 (0–25) U/mL	9.05 ± 4.26	9.41 ± 4.28	8.28 ± 4.17	0.128
CA199 (0–27) U/mL	14.83 ± 14.06	15.14 ± 15.71	14.18 ± 9.82	0.696
NSE (0–16.3) ng/mL	14.80 ± 5.66	14.61 ± 4.72	15.18 ± 7.30	0.563
Comorbidities, *n* (%)				
Impaired hepatic function	46 (30.1)	20 (19.2)	26 (53.1)	<0.001
Pulmonary infection	73 (47.7)	27 (26.0)	46 (93.9)	<0.001
Therapy, *n* (%)				
Corticosteroids	138 (90.2)	94 (90.4)	44 (89.8)	1.000
Immunoglobulin	49 (32.0)	21 (20.2)	28 (57.1)	<0.001
EEG, *n* (%)	83 (54.2)	52 (50)	31 (63.3)	0.164
MRI, *n* (%)	70 (45.8)	57 (54.8)	13 (26.5)	0.002

*Note*: IMV, invasive mechanical ventilation; AE, autoimmune encephalitis; NMDAR, N-methyl-D-aspartate receptor; MOG, myelin-oligodendrocyte glycoprotein; LGI 1, leucine-rich glioma-inactivated 1; GAD 65, glutamic acid decarboxylase antibody 65; GABABR, *γ*-aminobutyric acid type B receptor; CASPR2, contactin-associated protein-like 2; AMPAR, *α*-amino-3-hydroxy-5-methyl-4-isoxazolepropionic acid receptors; CBA, cytometric bead assay; CSF, cerebral spinal fluid; TPOAb, thyroid peroxidase antibody; TGAb, thyroglobulin antibody; FT3, free triiodothyronine; FT4, free thyroxine; TSH, thyrotropin; ICP, ICP, intracranial pressure; CRP, C-reactive protein; ESR, erythrocyte sedimentation rate; CEA, carcinoembryonic antigen; AFP, alpha fetal protein; CA125, carbohydrate antigen 125; NSE, neuron-specific enolase; MRI, agnetic resonance imaging; EEG, electroencephalography.

**Table 2:** Prediction factors for IMV in AE patients.

Intercept and variables	Prediction model	OR (95% CI)	*P*-value
	*β*		
Intercept	−7.927		
Gender	−1.388	**0.250 (0.101–0.616)**	**0.003**
Impaired hepatic function	0.983	**2.671 (1.014–7.038)**	**0.047**
Antibody subtype	−0.345	0.708 (0.306–1.638)	0.420
CSF CBA	0.278	1.321 (0.788–2.213)	0.291
Blood CBA	0.482	1.619 (0.996–2.630)	0.052
CSF pressure (80–180) mmH_2_O	0.009	**1.008 (1.001–1.015)**	**0.031**
CSF chloride (120–132) mmol/L	0.032	1.032 (0.952–1.120)	0.442
ESR (0–20) mm/hr	0.011	1.011 (0.988–1.034)	0.366
CRP (0–6) mg/L	0.009	1.009 (1.000–1.018)	0.054
NSE (0–16.3) ng/mL	0.087	**1.091 (1.014–7.038)**	**0.047**

*Note*: *β* is the regression coefficient. *Abbreviation*. IMV, invasive mechanical ventilation; AE, autoimmune encephalitis; CBA, cytometric bead assay; CSF, cerebral spinal fluid; ESR, erythrocyte sedimentation rate; CRP, C-reactive protein; NSE, neuron-specific enolase. Bold values represent *P* < 0.05.

## Data Availability

The data used to support the findings of this study are available from the corresponding author upon request.
